# Psychiatrists’ Experience of a Peer Support Group for Reflecting on Patient Suicide and Homicide: A Qualitative Study

**DOI:** 10.3390/ijerph192114507

**Published:** 2022-11-04

**Authors:** Millie Tamworth, Helen Killaspy, Jo Billings, Rachel Gibbons

**Affiliations:** 1 Division of Psychiatry, University College London, London W1T 7NF, UK; 2 Camden & Islington NHS Foundation Trust, London NW1 0PE, UK; 3 Royal College of Psychiatrists, London E1 8BB, UK

**Keywords:** psychiatrists, mental health practitioners, peer-support, postvention, suicide bereavement, qualitative research

## Abstract

There is a lack of support for mental health professionals who experience a patient suicide or homicide. This is despite a high likelihood of such an occurrence and the heavy professional and personal toll the experience can take. We conducted 15 interviews with members of a facilitated peer support group run for consultant psychiatrists who have experienced a patient homicide or suicide. Our interviews explored the trauma of the experience as well as the effectiveness of the group in helping the clinician heal. Our results echoed previous research that the experience can be profoundly traumatic. A professionally facilitated, consultant-only peer group specifically dedicated to suicide and homicide were the key components helping participants to process their grief. Mental health trusts should consider setting up facilitated peer support groups for clinicians who experience patient suicide or homicide.

## 1. Introduction

There is limited understanding of what kind of support is helpful for clinicians after a patient suicide or homicide. This is despite the fact that the impact of losing a patient in this way can be prolonged and profound [1]. In the field of suicidology, it has been suggested that coping with bereavement after a suicide can be complicated by higher levels of stigma and associations of blame [2]. In an effort to deepen our understanding of what support is needed, we conducted a qualitative evaluation of a peer support group for consultant psychiatrists who had experienced a suicide or homicide by a patient under their care.

In England, between 2008 and 2018, 1/3 of suicides were carried out by people under the care of mental health services, equating to 1271 suicides per year [3]. Typically, these patients were under the care of a multi-disciplinary team including a consultant psychiatrist, a psychologist, nursing staff, trainees and allied health professionals [4]. The total number of consultant psychiatrists in England as of September 2020 was 4452 [5]. Based on the yearly average of 1271 suicides, each psychiatrist has a one in three chance of losing a patient in this way every year. Put differently, over a 15-year career, the average consultant psychiatrist will experience five patient suicides although that number will vary depending on the area of specialty. Rates of homicide carried out by patients are lower, averaging 54 a year [3], but the personal and professional repercussions of such an event appear both more common and more severe. In a survey assessing the impact of patient homicide on forensic psychiatrists, 81% of respondents said the experience had impacted their mental health, 25% of which described it as ‘very significant’ [6].

The impact of patient suicide is more widely covered than homicide, due in part to its higher frequency. A recent systematic review outlined the different ways that psychiatrists are affected by patient suicide, whilst Mezey et al. carried out a survey looking at the impact of homicide on the care team [6,7]. Both papers highlight the emotional effect on the individual (such as guilt, shame, blame and anger) and the impact on their professional practice (such as taking on lower-risk patients, prescribing more antidepressants). The severity and duration of these impacts depends on several factors, including the psychiatrist’s personality traits, the nature of the therapeutic relationship they had with the patient and the manner of their death [7]. Sandford et al. found that the most significant risk factor for a negative impact was fear of blame or self-blame [7]. Previous research has also found feelings of stigma and persecution to be prevalent [6,8]. To that end, the psychiatrist’s experience echoes the wider literature on experiences of suicide and homicide bereavement.

To date there is little evidence as to what organisational and cultural factors may moderate the impact of a patient suicide or homicide on professionals. Sandford et al.’s study noted that informal support was deemed most helpful, but it was unclear whether formal support was considered inadequate or required more individualisation [7]. It has also been noted that the evidence base for what types of interventions are useful for those impacted by suicide more widely is underdeveloped [9].

Research on specific occupational groups exposed to suicide found that, in the case of ambulance workers, a combination of time pressures and a macho culture prevented people seeking support [10]. The study also found that there was limited opportunity for staff to process their own distress but when afforded the means to do so, staff supported one another. For psychiatrists, the likelihood of seeking support needs to be framed in the context of clinical responsibility. The death of their patient by suicide may implicate and stigmatise them in a way that is less the case for doctors working in other specialties [8].

Campaigns and initiatives such as the ‘zero-suicide policy’ differ in their capacity to motivate workforces, depending in part on implementation. Zero-suicide can be construed positively, as an aspirational goal underlying concrete objectives of delivering reliable and consistent healthcare [11]. Conversely, the same policy can be presented as an absolute target or key performance indicator. Such policies can be unhelpful in exaggerating the degree to which health professionals can prevent these kinds of tragic incidents and risks implying that a ‘failure to prevent’ a suicide equates to it being the healthcare professional’s fault [2,12]. In the wake of a traumatic death, the difficult emotions that arise and the fact that the patient is not there to speak for themselves, means clinical responsibility and blame can conflate [6]. In this context, seeking support can be challenging [12].

The group is run out of an NHS Trust in London and operates an open membership, meaning any consultant psychiatrist is welcome to attend regularly, intermittently or as a one-off. The group was founded 12 years ago by two consultant psychiatrists. Today it is facilitated by one of the group founders who is also a trained psychotherapist. Group membership has grown, and a typical session is now eight to ten people. Five to seven of these are ‘core’ (regular) members, one of whom is the group facilitator; the remaining, non-core participants might attend following a relevant experience. The group meets every three months face to face for two hours.

Group sessions are loosely based on the Balint model [13] and use a psychoanalytical framework. Consultants share their experience of the event and their reaction-described by the group facilitator as a “stereotyped pattern of response”-with the rest of the group. They then listen to other members discussing what they have shared. This ‘stepping back’ gives the presenter space to observe their case from an objective viewpoint, uncoloured by their own interpretations and judgements.

A facilitated conversation is then held by the group who consider and reflect on what they have heard. The intention is to bring together multiple perspectives, provide emotional understanding, containment, and an alternative narrative to self-recrimination. Through a combination of the group’s collective memory and members’ own experiences, the presenter’s experience is ‘metabolised’ within the group and ‘handed back’ in a manageable form.

Our research had three research objectives:

- To investigate the experiences of psychiatrists after a patient suicide or homicide.
- To investigate the ways in which the group may be helpful to its members and ways in which it may be improved.
- To understand key elements of the group structure and the role of the facilitator.

## 2. Methods

### 2.1. Study Design, Participants and Procedures

The study procedures were approved by the University College London Research Ethics Committee (Ref. 20423/001). Consultant psychiatrists who were attending or had previously attended the group were asked if they were happy to be contacted by the research team. Willing participants were sent the Participant Information Sheet and Consent Form. They returned an electronically signed consent form to the principal researcher (MT) before the interview. The topic guide (Supplementary Materials) for the interview was drafted by the research team in consultation with the Royal College of Psychiatrists’ working group on patient suicide and homicide, which comprises psychologists, psychiatrists, academics, psychotherapists, and family members with lived experience of losing someone to suicide. MT conducted all the interviews, most of which took place remotely via Microsoft Teams. All the interviews were audio recorded and transcribed verbatim.

### 2.2. Analysis

Our study adopted a reflexive thematic analysis approach, whereby researcher subjectivity is regarded as an analytic resource [14]. Three members of the research team (MT, JB, HK) conducted the coding and reflexive analysis. HK is a member of the peer support group and subsequently, we ensured the transcript excerpts selected for her input guaranteed the anonymity of the participating member. Participants had been made aware that HK was part of the research team and would have access to participant data and consent was given on this basis. HK did not partake in the interview process. The fourth research team member, RG, is a founder of this peer support group. RG participated in the interview process and therefore did not partake in the analysis. Transcripts were imported into NVivo Pro V12 and a preliminary coding frame was developed. MT conducted initial coding before allocating two transcripts each to HK and JB, who coded these independently. The team then met for further refinement into a coding framework. Coding was done collaboratively, consisting of an interactive process between data, interviewer, and research team. Our purpose was to develop a rich and nuanced reading of the data rather than seek consensus on meaning.

Reflexive thematic analysis is an ongoing and iterative process that does not lend itself well to the concept of data saturation [15]. Our decision about how many people to interview was a function of the finite nature and specificity of the sampling pool rather than a need to reach data saturation. Our results take into consideration and give the reader an indication of when perspectives were unanimous and when opinions varied. Consistent with the principles of reflexive thematic analysis [15], it would be nonsensical to cite frequencies of responses. The interview process in reflexive thematic analysis is necessarily open and evolving, and not every participant is asked the exact same question at the same point in the interview. Certain participants may choose to give certain examples, but this decision could be as much a function of the stage of the conversation as it is an indicator of the relative importance of the topic in question. Our intention in using reflexive thematic analysis was to capture the breadth of experiences and responses, a single point of view is as valid as one which is shared by many [14].

### 2.3. Reflexivity

Reflexivity is the process of actively recognising your own personal situation as the researcher and the impact this may have on data interpretation [16]. It enables the reader to consider the researcher’s perspective as a factor in how the research was constructed, and hence the validity of the analysis [17]. The research team had four authors, MT, JB, HK and RG. Each encompassed different career stages, clinical specialties and academic areas of interest.

MT is a mature student studying an MSc in Clinical Mental Health Sciences. She has research interests in experiences of vicarious trauma across different occupational settings. This subject matter was interesting to MT who sought to better understand society’s coping mechanisms for dealing with the pain of suicide-seemingly through silence or by turning away- and how this contributes to the trauma, isolation and experience of stigma for those left behind [10].

JB is a Consultant Clinical Psychologist with over 20 years of experience working in the NHS. An associate professor, she is an experienced academic in all areas of trauma and in the writing of qualitative research. HK is a consultant psychiatrist with expertise in psychosis. A professor with a background in quantitative and qualitative research, her areas of academic interest are services and interventions for people with complex mental health problems. She is also a member of the support group studied in this research. RG is a consultant psychiatrist, psychotherapist and Chair of the Patient Safety Group and Working Group on the Effect of Suicide and Homicide on Psychiatrists at the Royal College of Psychiatrists. She was one of the three founding members of the peer support group following early career experiences of losing patients to suicide. JB, HK and RG all have lived experience of patient suicide and/or homicide. 

### 2.4. Quality and Validity

The quality of this research was based on the Standards for Reporting Qualitative Research Framework (SRQR) [17]; conceptual and design thinking for thematic analysis which formed the foundation of our methodological approach [16]; and the Oxford Handbook of Qualitative Research [18].

Subjectivity lies at the heart of reflexive thematic analysis. Our aim was less to control bias than to explore the complexity of human experience and allow the reader to reflect on researcher perspective as part of their own conclusions [16]. Nevertheless, we have sought to be rigorous, transparent and trustworthy in the reporting of our findings [17], routinely reflecting on our assumptions, expectations and choices throughout the research process [19].

We contacted 19 past and present members out of a total of 50, 15 of whom agreed to be interviewed. Whilst results are not necessarily generalisable beyond the sample, the participants were sufficiently diverse in ethnic diversity, clinical specialty and experience for us to capture a range of views. Those interviewed included male and female members, the group founders, regular and infrequent attenders, former regular attendees and those who had attended only once.

## 3. Results

Eight females and seven males took part. Areas of specialty and membership status are presented in Table 1. Interviews took place between June and September 2021 with each lasting 45–60 min. Findings were organised into separate domains based on the research objectives. Within each domain, inductive themes were identified (Table 2). Our results use pseudonyms to ensure anonymity.

### 3.1. The Experience of Psychiatrists after a Patient Suicide or Homicide

#### 3.1.1. Responsibility

##### Uniqueness of the Consultant’s Role

The amount of responsibility held by consultants on behalf of the rest of the team is felt particularly acutely after a suicide or homicide. Consultants described feeling.

*“…isolated. You’re working very closely in a team; you feel very supported on the one hand, but you are the only consultant.”*

(Michael)

with an overwhelming sense of responsibility from being

*“…the only consultant psychiatrist in a team. [You] have unique experiences in having to be the certain decision-maker… Ultimately in these sorts of situations, if you go to Coroner’s Court, you’ll be the person that’s there. There are different expectations of you–of what you should know.”*

(Patricia)

##### Types of Responsibility

Participants described three different responsibilities after a patient suicide or homicide. These related to the team, clinical decision-making and the formal responsibilities following a serious incident.

**Team responsibility.** Most participants agreed that, after a serious incident, they felt inhibited by their position of responsibility within the team when it came to processing their own emotions. Participants often used the word ‘containment’ to describe a process of actively managing other team members’ anxiety and uncertainty through listening and facilitating or leading on decision making.

*“The role of the consultant is to contain a team that is under stress so that the team is functional at times of very high workload. I try to contain the distress of junior doctors and nurses when I can.”*

(Nina)

Peter described the challenge of having to healthily process his own emotions but in a safe and contained way.

*“Imagine going into a debrief and the consultant breaking down in front of everyone. It would make the whole team even more unsettled. You are expected, by virtue of your senior role, to keep things going…but there is a balance between this and showing your vulnerable side.”*

**Clinical responsibility.** Participants described how decisions around patient safety are often taken in a culture of unrealistic expectations about what is controllable. They described their role as that of a ‘super-hero’, a belief held sometimes by themselves and often by those around them, that they should always be able to save patients.

Comparing a severely unwell psychiatric patient to someone with terminal cancer, one participant pointed out that the idea that the psychiatric patient might die would be unthinkable to most people whereas this would be an accepted reality for the cancer patient. Another compared psychiatrists to ICU doctors:

*“I don’t think society expects them to save everyone. The approach to psychiatry is very different. We should have predicted everything.”*

(Nina)

Many found these expectations particularly challenging after a serious incident and described a process whereby clinical responsibility could easily be conflated with self-blame.

*“It’s very difficult to differentiate how much of it is your blame and how much of it is professional. Initially, my professional integrity felt threatened. Then personal things come in as well. And this is when things start to merge.”*

(Adam)

These feelings were rarely alleviated by the knowledge that, clinically, reasonable decisions had been taken.

*“On an intellectual level I know these things can happen. But some part of you, somewhere, believes that if we do all the right things, it won’t. I thought, ‘There must be something wrong with me.’”*

(Anne)

**Formal responsibilities.** As part of the investigation, psychiatrists are usually required to attend the Coroner’s Court. Generally, this experience was described as extremely challenging. The fact of being a witness rather than the accused rarely tallied with the experience, and the presence of a jury added to feelings of culpability.

*“It really feels like you are in court and someone will determine whether you are guilty or not guilty. Well, I mean, who else will have a jury unless you are accused of something?”*

(Jenny)

Many described the experience of being cross-examined by representatives of the deceased’s family, sometimes with no mediation from the judge, as very exposing.

*“The relatives, the solicitor of the relatives, they come and don’t always see you as the witness for information. Their attitude can be quite hostile.”*

(Peter)

#### 3.1.2. Support

Following a homicide or patient suicide, the support psychiatrists were offered depended on the organisation they were part of as well as factors such as resource constraints.

##### Attitudes towards Seeking Support

All interviewees valued receiving support after the incident, with informal support (such as from peers) being the most common. Regarding the acceptability of seeking support, the consensus was that, whilst it was getting easier to do so, this remained difficult.

*“It might not necessarily be a conscious ‘you’re weak if you get help’ because that goes against what we would be telling anybody else, but yeah, you should be fine and just manage these things.”*

(Michael)

Some participants described a ‘stiff upper lip’ culture in their NHS Trust where these kinds of incidents were considered part of the job and having a forum to discuss their impact was seen as “sitting around talking about it”. Conversely, the Trust affiliated with this group was seen as more open and progressive regarding consultant support by virtue of its hosting of the peer group. More recent consultants explained this had been helpful in overcoming any perceived ‘wrongness’ in needing support.

*“It was an utter relief to see people who have been consultants for 15 to 20 years and who are still so deeply affected by the loss of a patient … Pain is a human response and there’s nothing wrong with me.”*

(Anne)

##### The Support Available

Most psychiatrists had attended some kind of team debrief following an incident, though the form this took varied widely. Team debriefs were considered helpful in terms of feeling collegiate with the team who had experienced the tragedy, but many participants felt constrained in processing their own emotions at these meetings because of their role as emotional containers for the team, as detailed earlier in Section 3.1.1.

Other forms of support that participants mentioned included reflective practice groups, peer groups and Balint groups, again with variation in availability between employing organisations. Often policies about the support that was available contrasted with reality:

*“On paper you’re supposed to have a peer group, a supervisor, someone you can go to clinically … But the reality of the situation is you are so clinically inundated there’s no time to be accessing those sorts of support, and the people we are getting support from are themselves inundated and overspent. … personally, I felt trapped in the deep end.”*

(Anne)

One consultant explained how shortages of skilled facilitators meant sessions were often cancelled, whilst others remarked it could impact how they were run. One described a Balint group they had attended as “a 15 min presentation and discussion, nothing more than that”, whilst clinicians using support structures to meet CPD requirements was mentioned by several consultants. Asked whether they could have brought the subject of patient suicide to these other forums, it was thought theoretically possible but practically unwise. Many support structures that were described tended to focus on clinical decision-making but were not considered safe or appropriate places to discuss the consultant’s emotional response and experience of a patient suicide or homicide.

*“The support was there but it was … very procedural. It’s all very business-like and there’s no emotional connection … no sharing of that kind of level of emotional response to the work that we do.”*

(Michael)

### 3.2. The Value of the Group to Its Members

#### 3.2.1. Different Uses for Different People

Participants used the group in two key ways.

##### An Alternative Perspective

Participants with an interest or training in psychotherapy tended to show a particular curiosity about the acts of suicide and homicide and the human response to it. This was discussed in different ways but underlying each was a belief that, to truly process the experience and accompanying emotions, one must go beyond medical discourse and instead query human decision-making behind the taking of a life, be it one’s own or another’s. One member spoke of the common tendency to try to comprehend the act within the Western-medicine framework of ‘illness. They felt that, while removing the suicidal act from our understanding of ‘normal’ human behaviour may feel less painful, it prevents true understanding and healing.

*“Helping professions want to be seen as dealing with illness, not people acting out in a very destructive manner. So [the group] was about legitimising thinking negatively about suicide.”*

(Ben)

Many, not all, considered this psychoanalytical lens an important input to the wider grieving process.

The factor valued by all members was a separate and safe space where it is permitted to question *‘What if no one did anything wrong and this still happened*?’ and consider an alternative narrative to self-blame. Consultants described how, by stepping away from their everyday environment, they could distance themselves from artificial constructs such as omnipotence or the superhero narrative. In the context of a patient suicide or homicide, these constructs were contributing to the sense of self-blame and were prohibiting grief.

*“You don’t see the madness because you are part of it. And then you start to reflect… but you cannot reflect and question until you get space.”*

(Jane)

It was felt to be important that the space was dedicated solely to processing suicide and homicide experiences, enabling members to discuss subjects that might be too uncomfortable to raise in mainstream support environments.

*“Our natural tendency is to not want to go near it because it’s too distressing. People will, unless the group is about this and only this, talk about other things.”*

(Elizabeth)

##### Leaving It Behind

Some participants attended the group only a few times. These participants described how they used the group as a place to share the ownership of an experience, before depositing the pain as a form of closure.

*“Once I had processed it and thought about it a bit, I didn’t really want to keep talking and I just wanted to forget about it, to the extent that I could.”*

(Nick)

Those with less interest in psychotherapy still found value in the interpretative framework. Jane, a core member, described members’ different purposes in attending as a contributing dynamic to its workings:

*“I think some come because they just need to leave it with us. Putting it down and leaving it there and going away.”*

*Interviewer: “And that’s enough?”*

*“That’s enough. I think that’s a very common and even helpful, reasonable thing to do. It’s a gift to us and provides material to work with. It’s a gift to them because they can come in green and go out pink. They can just go and get on with things.”*

#### 3.2.2. A Three-Stage Journey: Shedding, Safety and Growth

Participants described a three-step process of healing encompassing the temporary shedding of their professional responsibilities, containment by group members and the opportunity for personal growth.

##### Shedding

The group invites its members to step outside the hierarchy of the medical profession and, in doing so, deconstruct their beliefs about responsibility. Core member Sally explained:

*“In being a consultant, a superhero is the role you have to enact even if you don’t feel it. When you come into this group, you don’t have to do that, it’s safe to take your hat off.”*

All participants agreed that shedding responsibilities was possible because the group is only open to peers (fellow consultants).

*“I try to contain the distress of junior doctors and nurses when I can. But I need to be contained as well … We cannot have the people I am supposed to contain in the same room as people tasked with containing me.”*

(Nina)

Participants highlighted two tensions to the group structure. First, other team members apart from the psychiatrist need support too. Second, the consultant-only aspect was described by the group facilitator as a “…a difficult balance, there’s something about the omnipotence of us trying to be omnipotent.” In other words, restricting the group to senior clinicians gives it an implied superiority that risks endorsing the superhero narrative they are trying to escape.

##### Safety

Participants described the confidential exchange of personal experience as key to feeling safe in the group. In the act of presenting, a consultant shares ownership of their experience and its accompanying grief.

*“The loneliness of the experience is shared. It is validated, it is acknowledged. The loneliness is not just yours, somebody else is owning it as well.”*

(Adam)

Having shared their account, the presenter listens to others discuss it. Members bring personal experiences as well as reflections from past group discussions, and newer participants begin to see a commonality of experience and feelings. Unanimously, members spoke of how important the sense of sameness was to feeling validated and contained.

*“There’s something quite shameful for clinicians in a death by suicide. So, I think that the most helpful thing was hearing from others, recognising feelings and normalising them.”*

(Laura)

##### Post-Traumatic Growth

All participants felt that attending the peer group sessions had enabled self-growth. Three main areas of self-growth were described.

**Regaining the capacity to think:** Participants reflected on how the opportunity for growth came once they felt safe in the group setting. With their emotions validated, they now had at their disposal a new framework for interpreting what happened, in which they were not cast as playing a central role.

*“The blame narrative does not disappear. However, you can reflect better on what your role is and not what you fantasise your role to be.”*

(George)

Within this safe space, there is a renewed ability to adopt a thinking mind. This was felt to be as much a function of the sense of separateness, safety and sameness as it is of the actual exchange with other members explored in the previous section.

*“We could only move when this absolute terror about suicide-hat we were to blame for it-could reduce. It was only through having the space to think about it that gradually curiosity emerged.”*

(Jenny)

**Changes in work behaviour:** Participants described a greater awareness of their daily environment. For some core members, the ability to continue working effectively in acute areas rests on regularly attending the group.

*“Without the group I don’t think I would be continuing, certainly not as healthily.”*

(Patricia)

One non-core member who worked in an inpatient ward described a new readiness to take time off and to work from home more regularly to help manage their tendency to overwork. The most consistent change outlined was in team communication, specifically breaking down hierarchies that impeded communication.

*“It certainly has changed how I function in terms of openness … and how we check in with each other on an ongoing basis. I was keen to break down this whole, you know, consultant superhero narrative. I don’t think it does anyone any good.”*

(Anne)

**Changes in clinical practice.** Regarding changes to clinical practice, seemingly diverse responses centred on an underlying intention that clinical decisions should not be influenced by fear of recrimination and blame.

*“… to not be scared to listen to your intuition. To practice in a way that feels right rather than in a way that’s guided by concerns that something awful is going to happen which will end your career.”*

(Patricia)

Many other consultants talked about prioritising instinct over ‘the training manual’ and allowing more nuanced risk formulation. Peter described this as.

*“… a case of how you think about the risks with the patient, which isn’t reflected on the medical notes, on the risk assessment tool, on what the expectation is from the Trust.”*

### 3.3. Group Structure and Facilitator

#### 3.3.1. Membership Structure

The group operates with open membership and has different types of members. Core members are those who attend regularly; non-core members attend episodically or as a one-off.

##### Core Members

At the time of writing, there are five to six core members who attend the group regularly, many of whom have a background in psychotherapy. Core members described their role as upholding an alternative framework of thought to the one brought by the consultant who is sharing. George, a core member, explained his purpose as

*“… bringing a different perspective to suicide, which steps away from something purely psychiatric—where we can get a little bit stuck in thinking … ‘What was the diagnosis? Was the medication the right thing?’-and towards … ‘What is [an] event like suicide like? What are the energies harnessed?”*

This sense of stepping outside the medical formulation and into questioning underlying responses to the experience was a consistent theme.

*“A key role of a core member is to provide the emotional containment so the other person can process; to investigate what the hooks are in that case that make the pain so unbearable and the loss so profound … It’s not a clinical learning, it’s something much more … [It’s] about the emotional structure of how we deal with this very difficult professional issue.”*

(Nina)

Another role was described as holding the collective memory of the group. By sharing the similarities of past cases, core members could show that the emotional experience is not unique.

*“You find yourself having similar sort [s] of explorations. The themes about responsibility and roles etc come up again and again.”*

(George)

As noted in Section 3.2.2., a sense of ‘sameness’ is critical to feeling validated and safe. Core members described experiencing ongoing reassurance from regularly attending the group and hearing other people’s cases. One explained how seeing reactions in other people helped them understand their own weak points better, which amounted to an iterative learning process.

*“You have the opportunity to digest your own episodes, again and again through new perspectives. Even if you don’t present, your own experience is still being re-evaluated.”*

(Adam)

##### Non-Core Members

At the time of writing, the group hosts two to three visiting members. Generally, we found that core members had stronger psychoanalytical interests than members who came less regularly. These differences were commented on by non-core members.

*“The people that attend regularly have been going for longest, [they] are more attuned to the implicit rules of the group [and] the styles of interpretation that are likely to land well.”*

(Nick)

A sense of “us and them”, particularly when first attending, was mentioned. One participant (now a core member) described his first visit as

*“… not easy. Relationships were already established. The way they were talking about things, the jargon, the facial expressions, the exchange of ideas… I thought, “Well, if I had to present a case, how would I feel?”*

(Adam)

However, this did not seem to impact the effectiveness of the group.

*“… there’s two subgroups within the group. It’s probably a little bit of an awkward mix but it doesn’t preclude it from being useful.”*

(Nick)

Whilst some visiting members described feeling safe reasonably quickly, it was rare that people had felt comfortable presenting their experience without having first attended as a listener.

#### 3.3.2. Facilitation: What’s Important

Participants were asked whether the group facilitator should be from outside the Trust and whether it was important they had psychoanalytical training. The current facilitator has knowledge of the Trust but does not currently work in it. All participants knew the facilitator in a professional capacity outside the group. This familiarity appeared more important than either of the above questions.

*“I have never thought about it their position within [the Trust], it feels so natural they are there.”*

(Adam)

*“Inside is better. The support you need is also about trust and understanding the institutional dynamics at play.”*

(Jenny)

Regarding psychoanalytical training, many of those personally inclined to that way of thinking answered in relation to the importance of employing a psychoanalytical lens.

*“Absolutely critical [a psychoanalytical lens]. It creates the framework in which difficult things can come to the surface, things that you know were not visible before.”*

(Peter)

*“It allows the group to be held and to keep the group going in a way that progresses and furthers that kind of psychoanalytic stream of conversation.”*

(Anne)

The skill and experience of the facilitator was recognised by all participants and was an important factor in their decision to attend. It was described by many participants as paramount to feeling safe:

*“I wouldn’t feel comfortable and contained if it was just a Joe Bloggs psychiatrist facilitator. I wouldn’t feel safe in that space.”*

(Michael)

*“I think [they] are very skilled in facilitating. I think without them it might feel different.”*

(Laura)

## 4. Discussion

### 4.1. Summary of Research Objective and Results

Our study had three research objectives:

- To investigate the experience of psychiatrists after a patient suicide or homicide
- To investigate the ways in which the consultant peer group may help its members and ways in which it may be improved
- To understand key elements of the group structure and the role of the facilitator

Our data revealed that a sense of fellowship is critical to allowing psychiatrists to first grieve and then grow after experiencing a patient suicide or homicide. It showed the impact of these events depends on several factors including the circumstances surrounding the suicide or homicide, the psychiatrist’s sense of their therapeutic alliance with the patient, the manner of the death and the approach taken in the subsequent investigation and coroner’s inquest. Our findings in this regard were broadly consistent with research conducted previously [7] although we note that research on the investigative and inquest experience is lacking. Despite the range of potential factors associated with our participants’ experiences, we identified common emotional responses of psychiatrists including feelings of acute guilt or shame. Within the peer group setting, this commonality becomes evident through the intimate act of sharing one’s own experience and through the collective history of the group shared by core members.

### 4.2. The Experiences of Psychiatrists after a Patient Suicide or Homicide

The important difference between this support group and other support structures was in providing separation from the conventional clinical framework of thought which, in the context of a suicide, can trap the consultant in a narrative of self-blame. Therefore, this support group was described as helping consultants in a way that other forms of support do not. It does so by being a space dedicated to the processing of the experience of suicide or homicide and by offering a different lens through which to view the experience.

Our participants described the difficulty presented by the superhero narrative that can be held by themselves and society. Whilst the superhero narrative is not confined to psychiatry; its falsity can be accentuated by the experience of a patient suicide or homicide. Gorkin has written about how the extent of a clinician’s belief in their omnipotence can shape their trauma after a suicide [20]. Expectations of clinical omnipotence was also reported in a recent meta-synthesis of 10 papers exploring the impact of patient suicide across doctors and nurses [21]. This paper noted differences in blame attribution between doctors and nurses. Doctors reported looking inwardly whereas nurses looked to external factors. Other research is less clear-cut about the personal impact of patient suicide on clinicians [22]. The varied findings alert us the individual nature of the experience and the multitude of factors that can determine how a suicide or homicide impacts a clinician [23]. This goes beyond personality to include contextual and environmental factors [7].

Plakun and Tillman describe a twin bereavement of personal and professional identity for psychiatrists after a patient suicide [24]. In our interviews, we explored the differences between a psychiatrists’ personal, professional and organisational self to gauge the different types of blame felt by participants. We found that many psychiatrists experienced a blurring of these domains that added to the challenge of grieving. One such context is the relationship between the psychiatrist and the patient’s family after a suicide or homicide. This can be very strained with families often needing to assign blame [25] at precisely the time when the psychiatrist’s personal self is seeking absolution. Currently there is no communication protocol for the family and medical team in the aftermath. More could be done to protect both the psychiatrist and family in these circumstances.

### 4.3. The Support Available

Previous research has shown that psychiatrists can be reluctant to seek support after a patient suicide or homicide [6,26]. Brooks et al. [27] have identified multiple barriers to doctors seeking help for mental health including anticipated negative career implications from either taking time off work or in disclosing their condition. The paper discusses doctors feeling shame, embarrassment, and a sense of weakness in the role reversal implied of becoming ‘the patient’.

The feedback from our participants echoed these findings but working in psychiatry introduces another layer of complexity, and the experience of a serious incident, yet another. Participants described psychiatry as having an unspoken cultural resistance toward professionals getting support for themselves, though the extent of this resistance depends on the specific trust and area of specialism. The silence over the issue of support captures the contradictory position of being in a profession which upholds the value of managing one’s mental health but not feeling able to seek it out for themselves. The sense of ‘not feeling entitled’ (to support) may become more prevalent when a psychiatrist graduates to a consultant role and their responsibilities-team and clinical-grow. We have discussed how in the case of a patient suicide or homicide it is common for psychiatrists to feel that they are to blame personally. A further issue then may be that the clinician fears that admitting the need for support is tantamount to an admission of professional guilt. More needs to be done to understand this tension. What our research does make clear is that there is no other situation where the responsibility of containment is so crucial to the wellbeing of your team yet currently, many psychiatrists do not feel able to access the tools that will enable them to do so.

Our study suggests that many other existing forms of support inadvertently magnify rather than challenge the collective silence around suicide. Our participants identified the group being only open to consultants as an important contributor to its effectiveness. This structure was described as enabling clinicians to escape the burden of the superhero narrative and find sanity by doing so. That said, not opening the group to a wider range of clinicians also risks endorsing the ‘superhero’ narrative they are trying to escape as well as perpetuating the myth of clinical omnipotence. Research on the potential pitfalls of peer groups should be noted [28] and degree of choice in support is important. Future research should compare peer groups to other interventions [29].

### 4.4. Opportunities for Growth

Post-traumatic growth refers to positive psychological change that may co-exist with significant psychological distress after a shattering of fundamental assumptions [30]. Many participants pointed to examples of growth, including more realistic appraisal of what they could impact and what they could not, and passing on these insights to clinicians moving into a consultant role. Changes to clinical practice was a complex topic in our interviews, but any such changes reported seemed to be the result of growth and entailed a subtle alteration of perspective in relation to responsibility and team interaction. However, growth only became possible once the consultant was released from feelings of blame and could reflect analytically on what had happened. Past research has described how experiencing patient suicide without getting the right support can lead to inhibited clinical decision-making and more defensive practice [7,31].

The need to be more accepting of the possibility of patient suicide has been discussed in past research [7,22]. This issue can be explored in relation to risk assessment-an area of change reported by many of our consultants who spoke of allowing greater intuition and focusing on the clinical encounter. The predictive value of suicide risk assessments is far from clear. One study of suicide risk assessment tools looked at 156 different types used in 85 NHS organisations. Of these, 85% were tick-box checklists which have been shown to be ineffective in predicting suicide [32,33]. Another study [34] looked at the effectiveness of risk assessment in discerning the odds of suicide across low-risk and high-risk inpatient populations. This meta-analysis found that over a 20-year period, the pooled odds ratio of suicide in the high-risk group compared with low-risk group was 7.1 but with an extreme level of between-study heterogeneity. The authors argued the heterogeneity limited any certainty that a similar result would be found across other inpatient populations. Our study gives warning that that distilling the complexity of suicide risk in this way can reinforce false notions about the ability of clinicians to be able to control and prevent such incidents. It also risks shutting down genuine communication between mental health professionals and the patient [35].

### 4.5. Strengths and Limitations

Little is known about what the most effective interventions are for supporting individuals who experience the death of a patient by suicide or a homicide by a patient under their care. This study adds to the research in the field and enriches overall understanding. Most group members were interviewed, enabling the accumulation of a wide range of perspectives from clinicians of different career stages, areas of psychiatry and genders. The qualitative approach taken enabled us to explore the “phenomenological complexity” of the experience [12]. We examined individual experiences of trauma and the effectiveness of this intervention in helping clinicians recover. Our conversations also explored other forms of support offered, and their respective merits and shortcomings.

However, this was a small-scale, qualitative study, and findings may not be transferrable to other settings. Additionally, whilst good understanding into why the group is only open to consultants was gained, more research is needed on the experiences of other team members and alternative support mechanisms that could be helpful. Finally, the study only interviewed participants of the peer group, which introduces self-selection bias. Whilst participating consultants were asked their views on the limitations of the group, the perspectives of those who chose not to attend the group are essential to fully understanding pitfalls.

### 4.6. Clinical Implications

Our findings demonstrate the potential benefits of providing a facilitated peer support group for consultant psychiatrists. Whilst this study focused on consultant psychiatrists there is no reason to consider that such groups would not be useful to other professional groups as well. Further research is needed to understand whether support groups that include a range of different professionals would be feasible or as well attended and effective as the group studied in this project.

Clearly this is not the only potential staff intervention needed following these kinds of serious incidents and other structures, such as receiving specific support and guidance to navigate the subsequent formal process that follow a patient suicide or patient perpetrated homicide are likely to be needed. Further research is needed to clarify the kinds of assistance and guidance staff may benefit from.

## 5. Conclusions

This study builds on previous research showing that the experience of a patient homicide or suicide can be profoundly distressing to psychiatrists and confirms the complexity of the grieving process in tragic situations such as these. Ensuring that clinicians have access to appropriate support is crucial and this study identified many benefits of a facilitated consultant psychiatrist peer support group. Our study has helped to expand the understanding of the underlying causes of this distress and how the structure and facilitation of a peer support group can address them. Further research is needed to investigate the effectiveness of this and other forms of support for clinicians and what kind of support works for whom and why. What we do know at this juncture is that actively supporting psychiatrists, by addressing the stigma they face and the sense of blame they feel, is extremely important for their mental health and well-being, and ultimately their capacity as professionals.

## Figures and Tables

**Table 1:** Demographic Information of participants.

Participant	Membership Status	Specialist Area
001	Present	Eating Disorders
002	Present	Rehabilitation Psychiatry
003	Present	Old Age Psychiatry
004	Present	Assessment
005	Present	General Adult Psychiatry
006	Present	Acute Care
007	Present	General Adult Psychiatry
008	Present	Acute Care
009	Present	Personality Disorder Unit
010	Past	Acute Care
011	Past	Acute Care
012	Past	Perinatal
013	Past	General Adult Psychiatry
013	Past	Psychiatric Intensive Care
015	Past	Early Intervention Services

**Table 2:** Themes and sub-themes by research objectives.

1. The Experience of Psychiatrists After a Patient Suicide or Homicide
1.1 Responsibility
1.1.1 Uniqueness of consultant’s role
1.1.2 Types of responsibility
1.1.2.1 Team
1.1.2.2 Clinical
1.1.2.3 Formal
1.2 Support
1.2.1 Attitudes towards seeking support
1.2.2 The support available
**2. The value of the group to its members**
2.1 Different uses for different people
2.1.1 An alternative perspective
2.1.2 Leaving it behind
2.2 A three-stage journey
2.2.1 Shedding
2.2.2 Safety
2.2.3 Post-traumatic growth
2.2.3.1 Regaining the capacity to think
2.2.3.2 Changes in work behaviour
2.2.3.3 Changes in clinical practice
**3. Group structure and the role of the facilitator**
3.1 Membership structure
3.1.1 Core members
3.1.2 Non-core members
3.2 Facilitation: what’s important?

## Supplementary Materials

The following supporting information can be downloaded at: https://www.mdpi.com/article/10.3390/ijerph192114507/s1, Interview Transcript.
