# Supplementary material for: Psychiatrists’ Experience of a Peer Support Group for Reflecting on Patient Suicide and Homicide: A Qualitative Study
Source: Int J Environ Res Public Health. 2022 Nov 4;19(21):14507. doi: 10.3390/ijerph192114507 (PMC9654625; doi:10.3390/ijerph192114507)
Supplement: Supplementary file 1 [file ijerph-19-14507-s001.zip › ijerph-1897371-supplementary.pdf]

Interview Topic Guide

- Perhaps we could start by you telling me a bit about you and your current role as a psychiatrist?

Their involvement with the group:

- I'd love to hear about you how you came to be involved with the group
- What were the issues that made you come to seek support in the first place?
- Can you give me a sense of the time interval between the event and your first coming to the group?
- At the time did you use support mechanisms alongside this one?
- What about the support mechanisms used today?

Their perspective on how the group is structured

- I'd like to understand your experience of the group structure. For example the group is only open to consultants, is this important?
- How well do you know the other consultants professionally and personally – was this a positive or negative for you?
- What about the position of the group facilitator? Is it important that it someone outside the trust?
- Is the discussion facilitated in a way you find helpful?
- Does the group meet regularly enough / for the right length of time?
- The membership ebbs and flows and the group is an open one. How do you feel about this? (and why)
- Over time has either has your experience of the group changed or your experience as a group member?
- Your facilitator is trained in psychotherapy. How important is this?
- The group is open to those who have lose patients through suicide and those who have had patients commit homicide and some have experienced both. What are your thoughts about this?
- Is there anything about how the group is structured or facilitated that you would consider changing?

Their experience of attending the group:

- I would like to explore how the experience of attending the group has impacted you personally. What impact has the group had on your experience of dealing with a patient suicide or homicide?
- If we think of you as having a personal, professional and organisational self. Which of these, if any, has the group helped?
- What was it like sharing your account?
- What was it like hearing someone elses?
- Did you ever feel triggered in taking part in the group?

Impact on professional self:

- How has the group impacted on you professionally?
- What, if anything, have you learned or taken away from attending the group?
- Has the group altered aspects of your clinical behaviour?
    - Making a risk assessment of the patient
    - How you communicate with your patient?
    - Any other aspect of clinical approach
- Can you grow as a clinician after an event such as this?

Support for consultants generally:

- Some research has indicated that clinicians can be profoundly impacted by traumatic events related to their patients but many don't take time off work. Why do you think this is?
- What do you think more generally about consultants attitude towards getting help?
- Do you think that mental health organisations' thinking about this has evolved over time?
- Does having a group have an effect on the wider organisation (raising awareness of issue etc)
- Anything else?
